# Direct Inference of Base-Pairing Probabilities with Neural Networks Improves Prediction of RNA Secondary Structures with Pseudoknots

**DOI:** 10.3390/genes13112155

**Published:** 2022-11-18

**Authors:** Manato Akiyama, Yasubumi Sakakibara, Kengo Sato

**Affiliations:** 1 Department of Biosciences and Informatics, Keio University, 3-14-1 Hiyoshi, Kohoku-ku, Yokohama 223-8522, Japan; 2 School of System Design and Technology, Tokyo Denki University, 5 Senju Asahi-cho, Adachi-ku, Tokyo 120-8551, Japan

**Keywords:** RNA secondary structure, deep learning, pseudoknots

## Abstract

Existing approaches to predicting RNA secondary structures depend on how the secondary structure is decomposed into substructures, that is, the *architecture*, to define their parameter space. However, architecture dependency has not been sufficiently investigated, especially for pseudoknotted secondary structures. In this study, we propose a novel algorithm for directly inferring base-pairing probabilities with neural networks that do not depend on the architecture of RNA secondary structures, and then implement this approach using two maximum expected accuracy (MEA)-based decoding algorithms: Nussinov-style decoding for pseudoknot-free structures and IPknot-style decoding for pseudoknotted structures. To train the neural networks connected to each base pair, we adopt a max-margin framework, called structured support vector machines (SSVM), as the output layer. Our benchmarks for predicting RNA secondary structures with and without pseudoknots show that our algorithm outperforms existing methods in prediction accuracy.

## 1. Introduction

The roles of functional non-coding RNAs (ncRNAs) in regulating transcription and guiding post-transcriptional modification have been recently shown to be critical in various biological processes, ranging from development and cell differentiation in healthy individuals to disease pathogenesis [1]. The well-established relationship between the primary sequence and structure of ncRNAs has motivated research aiming to elucidate the functions of ncRNAs by determining their structures.

Yet, methods for experimentally determining RNA tertiary structures utilizing X-ray crystal structure analysis and nuclear magnetic resonance (NMR) are costly and labor-intensive, thus restricting their application. Accordingly, researchers often carry out computational prediction of RNA secondary structures based on the analysis of base pairs comprising nucleotides joined by hydrogen bonds.

Computational approaches to RNA secondary structure prediction often utilize thermodynamic models (e.g., Turner’s nearest neighbor model [2,3]) that define characteristic substructures, such as base-pair stacking and hairpin loops. In computational approaches, the free energy of each type of substructure is first empirically determined by methods such as optical melting experiments [2]. Then, the free energy of RNA secondary structures can be estimated as the sum of the free energy of their substructures. Dynamic programming can then be used to determine the optimal secondary structure that minimizes free energy for a given RNA sequence. This approach is employed by RNAfold [4], RNAstructure [5] and UNAfold [6], among other tools.

As an alternative to experimental approaches, machine learning can be utilized to train scoring parameters based on the substructures constituting reference structures. This type of approach, as implemented in CONTRAfold [7,8], Simfold [9,10], ContextFold [11] and similar tools, has improved the accuracy of RNA secondary structure prediction. By integrating thermodynamic and machine-learning-based weighting approaches, MXfold avoided overfitting and achieved better performance than models based on either one alone [12]. Furthermore, interest in the use of deep learning for RNA secondary structure prediction is rapidly increasing [13,14,15]. MXfold2 used thermodynamic regularization to train a deep neural network so that the predicted folding score and free energy are as close as possible. This method showed robust prediction results in familywise cross validation, where the test dataset was structurally different from the training dataset.

Another important aspect of RNA secondary structure prediction is the choice of the decoding algorithm used to find the optimal secondary structure from among all possible secondary structures. Two classic decoding algorithms are the minimum free energy (MFE) algorithm, which is used in thermodynamic approaches, and the maximum likelihood estimation (MLE) algorithm, which is used in machine-learning-based approaches. These algorithms find a secondary structure that minimizes the free energy and maximizes the probability or scoring function, respectively. Another option is a posterior decoding algorithm based on the maximum expected accuracy (MEA) principle, which is known to be an effective approach for many high-dimensional combinatorial optimization problems [16]. As researchers usually evaluate the prediction of RNA secondary structures using base-pair-wise accuracy measures, MEA-based decoding algorithms utilize posterior base-pairing probabilities that can be calculated by the McCaskill algorithm [17] or the inside–outside algorithm for stochastic context-free grammars. CONTRAfold [18] and CentroidFold [19] both have MEA-based decoding algorithm implementations that successfully predict RNA secondary structures.

**Figure 1:**
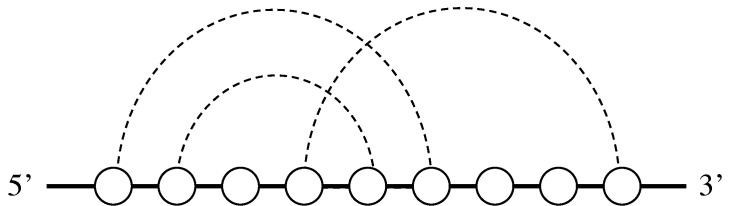
An example of pseudoknots.

Pseudoknots, an important structural element in RNA secondary structures, occur when at least two hydrogen bonds cross each other, and are typically drawn as two crossing arcs above a primary sequence (Figure 1).

Many RNAs, including rRNAs, tmRNAs and viral RNAs, form pseudoknotted secondary structures [20]. Pseudoknots are known to be involved in the regulation of translation and splicing as well as ribosomal frame shifting [21,22]. Furthermore, pseudoknots support folding into 3D structures in many cases [23]. Therefore, the impact of pseudoknots cannot be ignored in the structural and functional analysis of RNAs.

However, all of the aforementioned algorithms cannot consider pseudoknotted secondary structures owing to computational complexity. It has been proven that the problem of finding MFE structures including arbitrary pseudoknots is NP-hard [24,25]. Therefore, practically available algorithms for predicting pseudoknotted RNA secondary structures fall into one of the following two approaches: exact algorithms for a limited class of pseudoknots, such as PKNOTS [26], NUPACK [27,28], pknotsRG [29] and Knotty [30]; and heuristic algorithms that do not guarantee that the optimal structure will be found, such as ILM [31], HotKnots [32,33], FlexStem [34] and ProbKnot [35].

We previously developed IPknot, which enables fast and accurate prediction of RNA secondary structures with pseudoknots using integer programming [36,37]. IPknot adopts an MEA-based decoding algorithm that utilizes base-pairing probabilities combined with an approximate decomposition of a pseudoknotted structure into hierarchical pseudoknot-free structures. The prediction performance of IPknot is sufficient in terms of speed and accuracy compared with heuristic algorithms, and it is much faster than the exact algorithms.

Both thermodynamic approaches and machine-learning-based approaches depend on the method by which a secondary structure is decomposed into substructures, that is, the *architecture* (as referred to in [38]), to define their parameter space. Turner’s nearest neighbor model is the most well-studied architecture for predicting pseudoknot-free secondary structures, while the energy models for pseudoknotted secondary structures have not been sufficiently investigated, except for the Dirks–Pierce model [27,28] and the Cao–Chen model [39] for limited classes of pseudoknots. To our knowledge, an effective and efficient procedure to find a suitable architecture that can predict RNA secondary structures more accurately is still unknown.

Here, we propose a novel algorithm to directly infer base-pairing probabilities with neural networks instead of the McCaskill algorithm or the inside–outside algorithm, which both depend on the architecture of RNA secondary structures. Then, we employ the inferred base-pairing probabilities as part of a MEA-based scoring function for the two decoding algorithms: Nussinov-style decoding for pseudoknot-free structures, and IPknot-style decoding for pseudoknotted structures. To train the neural networks connected to each base pair, we adopt a max-margin framework, called structured support vector machines (SSVMs), as the output layer. We implement two types of neural networks connected to each base pair: bidirectional recursive neural networks (BiRNN) over tree structures and multilayer feedforward neural networks (FNN) with *k*-mer contexts around both bases in a pair. Our benchmarks for predicting RNA secondary structures with and without pseudoknots show that the prediction accuracy of our algorithm is superior to that of existing methods.

The major advantages of our work are summarized as follows: (i) our algorithm enables us to accurately predict RNA secondary structures with and without pseudoknots; (ii) our algorithm assumes no prior knowledge of the architecture that defines the decomposition of RNA secondary structures and thus the corresponding parameter space.

## 2. Methods

### 2.1. Preliminaries

The RNA sequence structure is modeled following the setup used by Akiyama et al. [12]. First, let Σ={A,C,G,U}, and let $\Sigma^{*}$ represent the set of all finite RNA sequences comprised of bases in Σ. For a sequence $x=x_{1}x_{2}\cdots x_{n}\in \Sigma^{*}$, let |x| represent the number of bases in *x*, referred to as the length of *x*. Let S(x) represent the set of all possible secondary structures formed by *x*. A secondary structure y∈S(x) can be described as a |x|×|x| binary-valued triangular matrix $y={\left(y_{ij}\right)}_{i<j}$, in which $y_{ij}=1$ if and only if bases $x_{i}$ and $x_{j}$ form a base pair linked by hydrogen bonds, including both canonical Watson–Crick base pairs (i.e., G-C and A-U) and non-canonical wobble base pairs (e.g., G-U).

### 2.2. MEA-Based Scoring Function

We employ the maximum expected accuracy (MEA)-based scoring function originally used for IPknot [36,37].

A secondary structure y∈S(x) is assumed to be decomposable into a set of pseudoknot-free substructures $\left(y^{\left(1\right)},y^{\left(2\right)},\ldots ,y^{\left(m\right)}\right)$ satisfying the following two conditions: (i) y∈S(x) can be decomposed into a mutually-exclusive set, that is, for 1≤i<j≤|x|, $\sum_{1\leq p\leq m}y_{ij}^{\left(p\right)}\leq 1$; and (ii) each base pair in $y^{\left(p\right)}$ can be pseudoknotted to at least one base pair in $y^{\left(q\right)}$ for ∀q<p. Each pseudoknot-free substructure $y^{\left(p\right)}$ is said to belong to *level p*. For each RNA secondary structure y∈S(x), there exists a positive integer *m* such that *y* is decomposable into *m* substructures without one or more pseudoknots (for more details, see the Supplementary Materials of [36]). Through the above decomposition, arbitrary pseudoknots can be modeled by our method.

First, to construct an MEA-based scoring function, we define a gain function of $\hat{y}\in S\left(x\right)$ with respect to the correct secondary structure y∈S(x) as follows:

(1) $$\begin{array}{cc} G_{\gamma }\left(y,\hat{y}\right)=\; & \gamma TP\left(y,\hat{y}\right)+TN\left(y,\hat{y}\right)\\ =\; & \sum_{i<j}\left[\gamma I\left(y_{ij}=1\right)I\left({\hat{y}}_{ij}=1\right)+I\left(y_{ij}=0\right)I\left({\hat{y}}_{ij}=0\right)\right]. \end{array}$$

Here, γ>0 represents a base-pair weight parameter, TN and TP represent the numbers of true negatives (non-base pairs) and true positives (base pairs), respectively, and I(condition) is an indicator function returning a value of either 1 or 0 depending on whether the condition is true or false.

The objective is to identify a secondary structure $\hat{y}$ that maximizes the expected value of the above gain function (1) under a given probability distribution over the space S(x) of pseudoknotted secondary structures:

(2) $$E_{y|x}\left[G_{\gamma }\left(y,\hat{y}\right)\right]=\sum_{y\in S(x)}G_{\gamma }\left(y,\hat{y}\right)P\left(y|x\right).$$

Here, P(y∣x) is the probability distribution of RNA secondary structures including pseudoknots. The γ-centroid estimator (2) has been proven to allow us to decode secondary structures accurately based on a given probability distribution [18].

Accordingly, the expected gain function (2) can be approximated as the sum of the expected gain functions for each level of pseudoknot-free substructures $\left({\hat{y}}^{\left(1\right)},\ldots ,{\hat{y}}^{\left(m\right)}\right)$ in the decomposed set of a pseudoknotted structure $\hat{y}\in S\left(x\right)$. Thus, a pseudoknotted structure $\hat{y}$ and its decomposition $\left({\hat{y}}^{\left(1\right)},\ldots ,{\hat{y}}^{\left(m\right)}\right)$ can be found that maximize the following expected value:

(3) $$\begin{array}{ccc} E_{y|x}\left[G_{\gamma }\left(y,\hat{y}\right)\right] & ≃ & \sum_{1\leq p\leq m}\sum_{y\in S(x)}G_{\gamma^{\left(p\right)}}\left(y,{\hat{y}}^{\left(p\right)}\right)\;P\left(y|x\right)\\ & = & \sum_{1\leq p\leq m}\sum_{i<j}\left[\left(\gamma^{\left(p\right)}+1\right)p_{ij}-1\right]{\hat{y}}_{ij}^{\left(p\right)}+C. \end{array}$$

Here, $\gamma^{\left(p\right)}>0$ is a weight parameter for level *p* base pairs and *C* is a constant that is independent of $\hat{y}$ (for the derivation, see the Supplementary Material of [18]). The base-pairing probability $p_{ij}$ represents the probability of base $x_{i}$ being paired with $x_{j}$. As seen in Section 2.4, we employ one of three algorithms to calculate base-pairing probabilities.

It should be noted that IPknot can be considered an extension of CentroidFold [18]. For the restricted case of a single decomposed level (i.e., m=1), the approximate expected gain function (3) of IPknot is equivalent to CentroidFold’s γ-centroid estimator.

### 2.3. Decoding Algorithms

#### 2.3.1. Nussinov-Style Decoding Algorithm for Pseudoknot-Free Structures

For the prediction of pseudoknot-free secondary structures, we find $\hat{y}$ that maximizes the expected gain (3) with m=1 under the following constraints on base pairs:

(4) $$\begin{array}{cccc} \; & \mathrm{maximize} & \; & \sum_{i<j}\left[\left(\gamma +1\right)p_{ij}-1\right]{\hat{y}}_{ij} \end{array}$$

(5) $$\begin{array}{cccc} \; & \mathrm{subject to} & \; & \left\{\sum_{j=1}^{i-1}y_{ji}+\sum_{j=i+1}^{\left|x\right|}y_{ij}\right\}\leq 1\;\left(1\leq \forall i\leq |x|\right), \end{array}$$

(6) $$\begin{array}{cccc} \; & \; & & y_{ij}+y_{kl}\leq 1\;\left(1\leq \forall i<\forall k<\forall j<\forall l\leq |x|\right). \end{array}$$

The constraint defined by Equation (5) means that each base $x_{i}$ can be paired with at most one base. The constraint defined by Equation (6) disallows pseudoknot.

This integer programming (IP) problem can be solved by dynamic programming as follows, similar to the Nussinov algorithm [40],

(7) $$\begin{array}{cc} M_{i,j} & =\max\left\{\begin{array}{c} M_{i+1,j}\\ M_{i,j-1}\\ M_{i+1,j-1}+\left(\gamma +1\right)p_{ij}-1\\ \max_{i<k<j}M_{i,k}+M_{k+1,j} \end{array}\right., \end{array}$$

and then tracing back from $M_{1,|x|}$.

#### 2.3.2. IPknot-Style Decoding Algorithm for Pseudoknotted Structures

Maximization of the approximate expected gain (3) can be solved as the following IP problem:

(8) $$\begin{array}{cccc} \; & \mathrm{maximize} & \; & \sum_{1\leq p\leq m}\sum_{i<j}\left[\left(\gamma^{\left(p\right)}+1\right)p_{ij}-1\right]{\hat{y}}_{ij}^{\left(p\right)} \end{array}$$

(9) $$\begin{array}{cccc} \; & \mathrm{subject to} & \; & \sum_{1\leq p\leq m}\left\{\sum_{j=1}^{i-1}y_{ji}^{\left(p\right)}+\sum_{j=i+1}^{\left|x\right|}y_{ij}^{\left(p\right)}\right\}\leq 1\;\left(1\leq \forall i\leq |x|\right),\\ \; & \; & & y_{ij}^{\left(p\right)}+y_{kl}^{\left(p\right)}\leq 1 \end{array}$$

(10) $$\begin{array}{cccc} \; & \; & & \;(1\leq \forall p\leq m,\;1\leq \forall i<\forall k<\forall j<\forall l\leq |x|),\\ \; & \; & & \sum_{i<k<j<l}y_{ij}^{\left(q\right)}+\sum_{k<i^{\prime }<l<j^{\prime }}y_{i^{\prime }j^{\prime }}^{\left(q\right)}\geq y_{kl}^{\left(p\right)} \end{array}$$

(11) $$\begin{array}{cccc} \; & \; & & \;(1\leq \forall q<\forall p\leq m,\;1\leq \forall k<\forall l\leq |x|). \end{array}$$

Note that Equation (3) requires the consideration of only base pairs $y_{ij}^{\left(p\right)}$ with base-pairing probabilities $p_{ij}$ being greater than $\theta^{\left(p\right)}=1/\left(\gamma^{\left(p\right)}+1\right)$. The constraint defined by Equation (9) means that each base $x_{i}$ can be paired with, at most, one base. The constraint defined by Equation (10) disallows pseudoknots within the same level *p*. The constraint defined by Equation (11) ensures that each level-p base pair is pseudoknotted to at least one base pair at each lower level q<p. We set m=2, which is IPknot’s default setting. This suggests that the predicted structure can be decomposed into two pseudoknot-free secondary structures.

### 2.4. Inferring Base-Paring Probabilities

Our scoring function (3) described in Section 2.2 is calculated by using base-pairing probabilities $p_{ij}$. In this section, we introduce two approaches for computing base-pairing probabilities. The first approach is a traditional one that is based on the probability distribution of RNA secondary structures, e.g., the McCaskill model [17] for pseudoknot-free structures and its extension to pseudoknotted structures, e.g., the Dirks–Pierce model [27,28]. The second approach proposed in this paper directly calculates base-pairing probabilities using neural networks.

#### 2.4.1. Traditional Models for Base-Pairing Probabilities

The base-pairing probability $p_{ij}$ is defined as

(12) $$p_{ij}=\sum_{y\in S(x)}I\left(y_{ij}=1\right)P\left(y|x\right)$$

from a probability distribution P(y∣x) over a set S(x) of secondary structures with or without pseudoknots.

For predicting pseudoknot-free structures, the McCaskill model [17] can be mostly used as P(y∣x) combined with the Nussinov-style decoding algorithm described in Section 2.3.1. The computational complexity of calculating Equation (12) for the McCaskill model is $O(|x{|}^{3})$ for time and $O(|x{|}^{2})$ for space when using dynamic programming. This model was implemented previously as CentroidFold [18,19].

For predicting pseudoknotted structures, we can select P(y∣x) from among several models. A naïve model could use the probability distribution *with* pseudoknots as well as Equation (2) in spite of high computational costs, e.g., the Dirks–Pierce model [27,28] for a limited class of pseudoknots, with a computational complexity of $O(|x{|}^{5})$ for time and $O(|x{|}^{4})$ for space. Alternatively, we can employ a probability distribution *without* pseudoknots for each decomposed pseudoknot-free structure, such as the McCaskill model. Furthermore, to increase the prediction accuracy, we can utilize a heuristic algorithm with iterative refinement that refines the base-pairing probability matrix from the distribution without pseudoknots. See [36] for more details. These three models were implemented in IPknot [36].

#### 2.4.2. Neural Network Models

In this research, we propose two neural network architectures for calculating base-pairing probabilities instead of the probability distribution over all RNA secondary structures.

**Figure 2:**
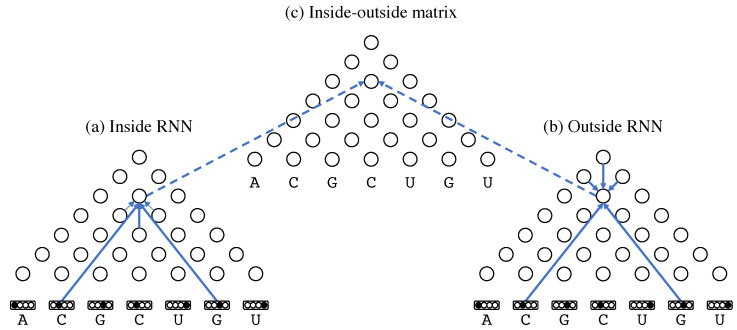
A bidirectional recursive neural network for calculating base-pairing probabilities. A set of four dots above each base represents the one-hot representation of the base. Each circle indicates a network layer with 80 hidden nodes. Each solid arrow indicate a connection between layers along grammatical trees derived from the RNA grammar. Each dashed arrow represents a connection that aggregates the inside and outside layers to output base-pairing probabilities.

The first architecture is the bidirectional recursive neural network (BiRNN) over tree structures as shown in Figure 2. Stochastic context-free grammars (SCFG) can model RNA secondary structure without pseudoknots [7,41]. The layers of BiRNN over the tree structure are connected along grammatical trees derived from SCFG that models RNA secondary structures. The BiRNN consists of three matrices—(a) the inside RNN matrix, (b) the outside RNN matrix and (c) the inside–outside matrix—for outputting base-pairing probabilities, each of whose elements contain a network layer (indicated by circles in Figure 2) with 80 hidden nodes. Each layer in the inside or outside matrix is recursively calculated from connected source layers as in the inside or outside algorithm, respectively, for stochastic context-free grammars (SCFG). The ReLU activation function is applied before being input to each recursive node. The base-pairing probability at each position is calculated from the corresponding layers in the inside and outside matrices with the sigmoid activation function. Our implementation of BiRNN assumes a simple RNA grammar

$$S\to aS\hat{a}|aS|Sa|SS|\epsilon ,$$

where a∈Σ, *a* and $\hat{a}$ represent the paired bases, S represents the start non-terminal symbol, and ϵ represents the empty string.

**Figure 3:**
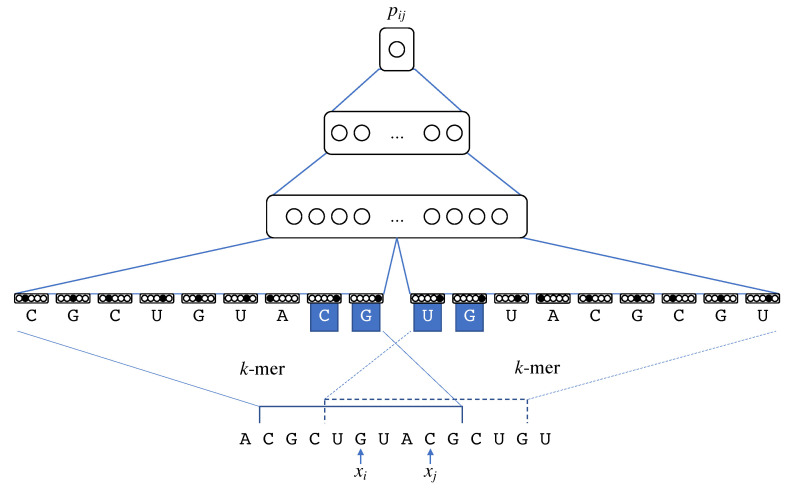
A feedforward neural network with k(=9)-mer contexts around $x_{i}$ and $x_{j}$ used to calculate the base-pairing probability $p_{ij}$. The end-of-loop nodes of the highlighted nucleotides are activated because they are beyond the paired bases.

The second architecture employs a simple multilayer feedforward neural network (FNN). To calculate the base-pairing probability $p_{ij}$, a FNN receives as input two *k*-mers around the *i*-th and *j*-th bases as shown in Figure 3.

Each base is encoded by the one-hot encoding of nucleotides and an additional node that indicates the end of the loop, which should be active for $x_{l}$ s.t. l≥j in the left *k*-mer around $x_{i}$ or $x_{l}$ s.t. l≤i in the right *k*-mer around $x_{j}$. This encoding can be expected to embed the length of loops and the contexts around the openings and closings of helices. We set k=81 for the *k*-mer context length default (for more details, see Section 3.4). We then construct two hidden layers consisting of 200 and 50 nodes, respectively, with the ReLU activation function and one output node with a sigmoid activation function to output base-pairing probabilities.

Note that the FNN model depends on no assumption of RNA secondary structures, while the BiRNN model assumes an RNA grammar that considers no pseudoknots. Instead, the FNN model can take longer contexts around each base pair into consideration by using longer *k*-mers.

### 2.5. Learning Algorithm

We optimize the network parameters λ by using a max-margin framework called a structured support vector machine (SSVM) [42]. For a training dataset $D={\left\{\left(x^{\left(k\right)},y^{\left(k\right)}\right)\right\}}_{k=1}^{K}$, where $x^{\left(k\right)}$ represents the *k*-th RNA sequence and $y^{\left(k\right)}\in S\left(x^{\left(k\right)}\right)$ represents the correct secondary structure of the *k*-th sequence $x^{\left(k\right)}$, we identify a λ that minimizes the objective function

(13) $$L\left(\lambda \right)=\sum_{(x,y)\in D}\left(\max_{\hat{y}\in S\left(x\right)}\left[f\left(x,\hat{y}\right)+\Delta \left(y,\hat{y}\right)\right]-f\left(x,y\right)\right),$$

where f(x,y) is the scoring function of RNA secondary structure y∈S(x) for a given RNA sequence $x\in \Sigma^{*}$, that is, Equation (4) for Nussinov-style decoding or Equation (8) for IPknot-style decoding. Here, $\Delta (y,\hat{y})$ is a loss function of $\hat{y}$ for *y* defined as

(14) $$\begin{array}{cc} \Delta (y,\hat{y})= & \delta^{\mathrm{FN}}\times \left(\#\mathrm{of}\mathrm{false}\mathrm{negative}\mathrm{base}\mathrm{pairs}\right)\\ \; & +\delta^{\mathrm{FP}}\times \left(\#\mathrm{of}\mathrm{false}\mathrm{positive}\mathrm{base}\mathrm{pairs}\right)\\ = & \delta^{\mathrm{FN}}\sum_{i<j}I\left(y_{ij}=1\right)I\left({\hat{y}}_{ij}=0\right)\\ \; & +\delta^{\mathrm{FP}}\sum_{i<j}I\left(y_{ij}=0\right)I\left({\hat{y}}_{ij}=1\right), \end{array}$$

where $\delta^{\mathrm{FN}}$ and $\delta^{\mathrm{FP}}$ are tunable hyperparameters that can control the trade-off between sensitivity and specificity in learning the parameters. By default, we used $\delta^{\mathrm{FN}}=\delta^{\mathrm{FP}}=0.1$. In this case, the first term of Equation (13) can be calculated using the Nussinov-style decoding algorithm or the IPknot-style decoding algorithm modified by loss-augmented inference [42].

To minimize the objective function (13), stochastic subgradient descent (Algorithm 1) or one of its variants can be applied. We can calculate the gradients with regard to the network parameters λ for the objective function (13) using the gradients with regard to $p_{ij}$ by the chain rule of differentiation. This means that the prediction errors occurred through the decoding algorithm backpropagating to the neural network that calculates base-pairing probabilities through the connected base pairs.
**Algorithm 1** The stochastic subgradient descent algorithm for structured support vector machines (SSVMs); η>0 is the predefined learning rate.1:initialize $\lambda_{k}$ for all $\lambda_{k}\in \lambda$2:**repeat**3:  **for all** (x,y)∈D **do**4:    $\hat{y}\leftarrow \arg\max_{\hat{y}}\left[f\left(x,\hat{y}\right)+\Delta \left(y,\hat{y}\right)\right]$5:    **for all** $\lambda_{k}\in \lambda$ **do**6:      $\lambda_{k}\leftarrow \lambda_{k}-\eta \left(\gamma +1\right)\sum_{i<j}\frac{\partial p_{ij}}{\partial \lambda_{k}}\left({\hat{y}}_{ij}-y_{ij}\right)$7:    **end for**8:  **end for**9:**until** all the parameters converge

## 3. Results

### 3.1. Implementation

Our algorithm is implemented as the program Neuralfold, which is short for the neural network-based RNA folding algorithm. We employ Chainer [43] for the neural networks and the Python linear programming solver PuLP [44]. The source code for this implementation is available at https://github.com/keio-bioinformatics/neuralfold/, (accessed on 27 September 2022).

### 3.2. Datasets

We evaluated our algorithm with the Nussinov-style decoding algorithm for predicting pseudoknot-free RNA secondary structures using four datasets, TrainSetA, TestSetA, TrainSetB and TestSetB, which were established by [45].

TrainSetA and TestSetA are literature-based datasets [7,9,10,41,46] that were constructed to ensure sequence diversity. TrainSetA contains SSU and LSU domains, SRP RNAs, RNase P RNAs and tmRNAs comprising 3166 total sequences spanning 630,279 nt, with 333,466 forming base pairs (47.9%). The sequence lengths range from 10 to 734 nt, with an average length of 199 nt. TestSetA includes sequences from eight RNA families: 5S rRNA, group I and II introns, RNase P RNA, SRP RNA, tmRNA, tRNA, and telomerase RNA. TestSetA contains 697 sequences, with 51.7% of their bases forming base pairs. The sequence length ranges from 10 to 768 nt, with an average length of 195 nt. We excluded a number of sequences that contain pseudoknotted secondary structures in the original data sources from TestSetA. Thus, 593 sequences were selected as TestSetA.

TrainSetB and TestSetB, which contain 22 families with 3D structures [38], were assembled from Rfam [47]. TrainSetB and TestSetB include sequences from Rfam seed alignments with no more than 70% shared identity between sequences. TrainSetB comprises 22 RNA families, and its specific composition is 145.8S rRNAs, 18 U1 spliceosomal RNAs, 45 U4 spliceosomal RNAs, 233 riboswitches (from seven different families), 116 cis-regulatory elements (from nine different families), 3 ribozymes and a single bacteriophage pRNA. TrainSetB was constructed by selecting sequences dissimilar to those in TestSetB. TrainSetB contains 1094 sequences, including 112,398 nt in all, of which 52,065 bases (46.3%) formed base pairs. The sequence length is in the range of 27 to 237 nt with an average length of 103 nt. TrainSetB contains 4.3% noncanonical base pairs. TestSetB also consists of the same 22 RNA families as TrainSetB, TestSetB contains 430 sequences, including 52,097 nt in all, of which 22,728 bases (43.6%) form base pairs. The sequence length is in the range of 27 to 244 nt, with an average length of 121 nt. TestSetB contains 8.3% noncanonical base pairs.

We also evaluated our algorithm with the IPknot-style decoding algorithm for predicting pseudoknotted RNA secondary structures on two datasets. The first dataset is called the pk168 dataset [48], which was compiled from PseudoBase [20]. This dataset includes 16 categories of 168 pseudoknotted sequences with lengths <140 nt.

The second dataset is called RS-pk388, originally established by [36]. This dataset was obtained from the RNA STRAND database and contains 388 non-redundant sequences with lengths between 140 and 500 nt.

### 3.3. Prediction Performance

We evaluated the accuracy of RNA secondary structure predictions based on sensitivity (SEN) and positive predictive value (PPV) as follows:

$$SEN=\frac{TP}{TP+FN},\;PPV=\frac{TP}{TP+FP}.$$

Here, TP, FP and FN represent the numbers of true positives (i.e., the correctly predicted base pairs), false positives (i.e., incorrectly predicted base pairs), and false negatives (i.e., base pairs in the correct structure that were not predicted), respectively. As a balanced measure of SEN and PPV, we utilized their *F*-value, which is defined as their harmonic mean:

$$F=\frac{2\times SEN\times PPV}{SEN+PPV}.$$

We conducted computational experiments on the datasets described in the previous section using the Nussinov-style decoding algorithm with the McCaskill and neural network models as well as the BiRNN and FNN models. We employed CentroidFold as the Nussinov decoding algorithm with the McCaskill model. We performed experiments on TestSetB using the parameters trained from TrainSetB. As shown in Table 1, the neural network models achieved better accuracy compared with the traditional model. Hereafter, we adopt the FNN model with *k*-mer contexts as the default Neuralfold model since it yielded better prediction accuracy in this experiment.

The other computational experiments on the pseudoknotted dataset were conducted using the IPknot-style decoding algorithm with the McCaskill model with and without iterative refinement and with the Dirks–Pierce model as well as using Neuralfold with the FNN model. Table 2 shows that the feedforward neural network (FNN) model with 10-fold cross validation is comparable to IPknot with the Dirks–Pierce model for pseudoknots but superior to the McCaskill model both with and without iterative refinement.

Table 3 shows the computation time for of the following sequences, which vary in length: PKB229 and PKB134 in the pk168 dataset; ASE_00193, CRW_00614 and CRW_00774 in the RNA STRAND database [49].

This shows that the computation time for predicting a pseudoknotted secondary structure using the FNN model is comparably fast to IPknot with the Dirks–Pierce model.

### 3.4. Effects of Context Length

**Figure 4:**
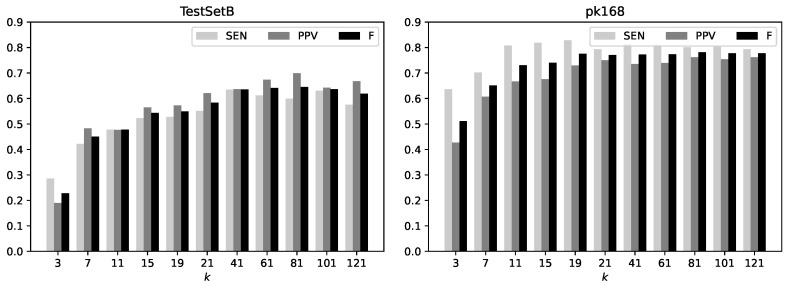
The accuracy of the FNN model with different lengths of *k*-mers on the TestSetB dataset (**left**) and the pk168 dataset (**right**). SEN, sensitivity; PPV, positive predictive value; *F*, the *F*-value based on SEN and PPV.

We evaluated the prediction accuracy obtained with the FNN model on the TestSetB and pk168 datasets for several lengths of *k*-mers input to neural networks. The accuracy as measured by SEN, PPV, and their *F*-value for different *k*-mer lengths k={3,7,11,15,19,21,41,61,81,101,121} is summarized in Figure 4. This analysis indicates that the accuracy is essentially maximized when the *k*-mer length is 81, and the difference in the accuracy for k≥81 is negligible.

### 3.5. Comparison with Previous Methods for Prediction of Pseudoknot-Free Secondary Structures

**Figure 5:**
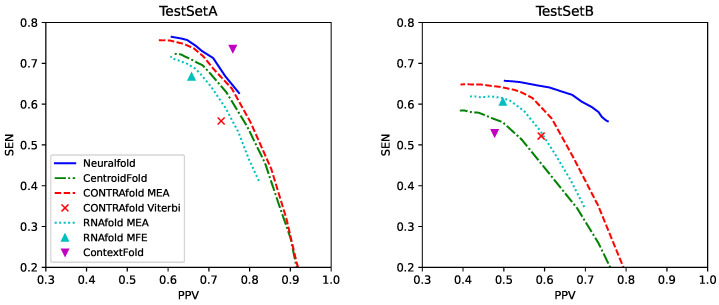
Positive predictive value–sensitivity (PPV–SEN) plots comparing our algorithm with competitive methods on TestSetA (**Left**) and TestSetB (**Right**).

We compared our algorithm with previous methods for predicting pseudoknot-free RNA secondary structures including CentroidFold [18,19], CONTRAfold [7,8], RNAfold in the Vienna RNA package [4] and ContextFold [29]. For the posterior decoding methods with the trade-off parameter γ in Equation (4), we used $\gamma \in \{2^{n}|n\in Z,\;-5\leq n\leq 10\}$. We performed secondary structure prediction on TestSetA with parameters trained on TrainSetA as well as prediction on TestSetB with the parameters trained on TrainSetB. The PPV–SEN plots for each method shown in Figure 5 indicate that our algorithm accurately predicts pseudoknot-free secondary structures in the datasets including famlilies similar with the training datasets.

On the other hand, to investigate the generalization ability of our method, another experiment in which our method was trained on TrainSetB and evaluated for accuracy on TestSetA showed that our method had very low accuracy (SEN=0.232, PPV=0.160, and F=0.189), which suggests that our method is severely overfitted.

### 3.6. Comparison with Alternative Methods for Predicting Pseudoknotted Secondary Structures

**Figure 6:**
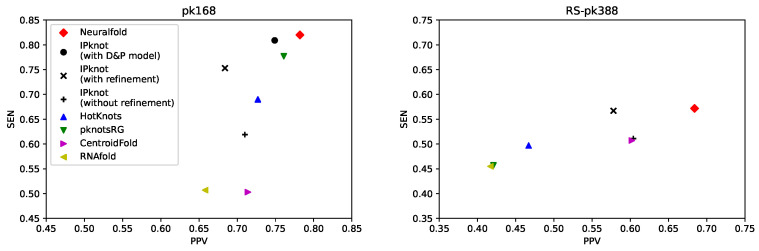
Positive predictive value–sensitivity (PPV–SEN) plots comparing our algorithm with competitive methods on the pk168 dataset (**Left**) and the RS-pk388 dataset (**Right**). For the pk168 dataset, we set $\gamma^{\left(1\right)}=1$, $\gamma^{\left(2\right)}=3$ for Neuralfold; $\gamma^{\left(1\right)}=2$, $\gamma^{\left(2\right)}=4$ for IPknot with the Dirks–Pierce (D&P) model; $\gamma^{\left(1\right)}=2$, $\gamma^{\left(2\right)}=16$ for IPknot with/without refinement; γ=2 for CentroidFold. For the RS-pk388 dataset, we set $\gamma^{\left(1\right)}=1$, $\gamma^{\left(2\right)}=3$ for Neuralfold; $\gamma^{\left(1\right)}=2$, $\gamma^{\left(2\right)}=2$ for IPknot without refinement; $\gamma^{\left(1\right)}=1$, $\gamma^{\left(2\right)}=1$ for IPknot with refinement; γ=2 for CentroidFold.

We also compared our algorithm with competing methods for predicting pseudoknotted secondary structures, including IPknot [36], HotKnots [32,33], and pknotsRG [29], as well as methods for predicting pseudoknot-free secondary structures, including CentroidFold [19] and RNAfold [4]. Neuralfold performed 10-fold cross validation on the pk168 and RS-pk388 datasets. Figure 6 shows PPV–SEN plots for each method, indicating that our algorithm works accurately on pseudoknotted datasets.

## 4. Discussion

We propose a novel algorithm for directly inferring base-pairing probabilities with neural networks that enables us to predict RNA secondary structures accurately. Sato et al. [36] previously proposed an iterative algorithm that refines the base-pairing probabilities calculated by the McCaskill algorithm so as to be appropriate for pseudoknotted secondary structure prediction. The direct inference of base-pairing probabilities with neural networks is an approach similar to the iterative refinement algorithm in the sense that both directly update base-pairing probabilities, and the IPknot-style decoding algorithm then uses the base-pairing probabilities. Although the iterative refinement algorithm can improve the prediction accuracy of IPknot to some extent, it should be noted that this is an ad hoc algorithm, as there is no theoretical guarantee of improvement. Meanwhile, the neural networks that infer base-pairing probabilities are trained on given reference secondary structures by the max-margin framework, meaning that we can theoretically expect that the neural network models improve the secondary structure prediction. Indeed, Table 2 shows that our algorithm achieved not only better accuracy than the iterative refinement algorithm, but is also comparable to that of the Dirks–Pierce model, which can calculate exact base-pairing probabilities for a limited class of pseudoknots.

Recently, several methods for predicting RNA secondary structure using deep learning were proposed [13,14,15]. Although most of them use deep learning to compute N×N matrices (*N* is the sequence length), which can be regarded as base-pairing probability matrices, they do not directly address the constraints that the RNA secondary structure must satisfy (e.g., Equations (5) and (6) for pseudoknot-free structures, and Equations (9)–(11) for pseudoknotted structures). On the other hand, MXfold2 [14] combines the Zuker-style dynamic programming [50] and deep learning to handle the constraints that pseudoknot-free RNA secondary structures must satisfy. UFold [15] predicts RNA secondary structure including pseudoknots using post-processing by linear programming, but does not directly address constraints on RNA secondary structure including pseudoknots when training deep learning models to predict base-pairing probabilities. By combining IPknot-style decoding with the max-margin training, the proposed Neuralfold can directly handle the constraints (9)–(11) that pseudoknotted RNA secondary structure must satisfy, not only when predicting secondary structures, but also when training deep learning models.

It has been pointed out that RNA secondary structure prediction based on machine learning and deep learning is prone to overfitting due to bias in the training data [14,45]. Several methods have been proposed to alleviate overfitting, such as using ensembles of multiple models [13], and integration with thermodynamic models [14]. UFold, on the other hand, employed artificially generated sequences and their predicted secondary structures for data augmentation, which were then used as additional training data to relax overfitting due to bias in the training data [15]. Our proposed method does not provide a strategy to counteract such overfitting and is therefore unsatisfactory at predicting sequences of families that are structurally distant from the training data, as shown in the results. However, by utilizing the ensembles of multiple models, as in SPOT-RNA, and the data augmentation strategy, as in UFold, it is expected to address to some extent the overfitting caused by bias in the training data.

**Figure 7:**
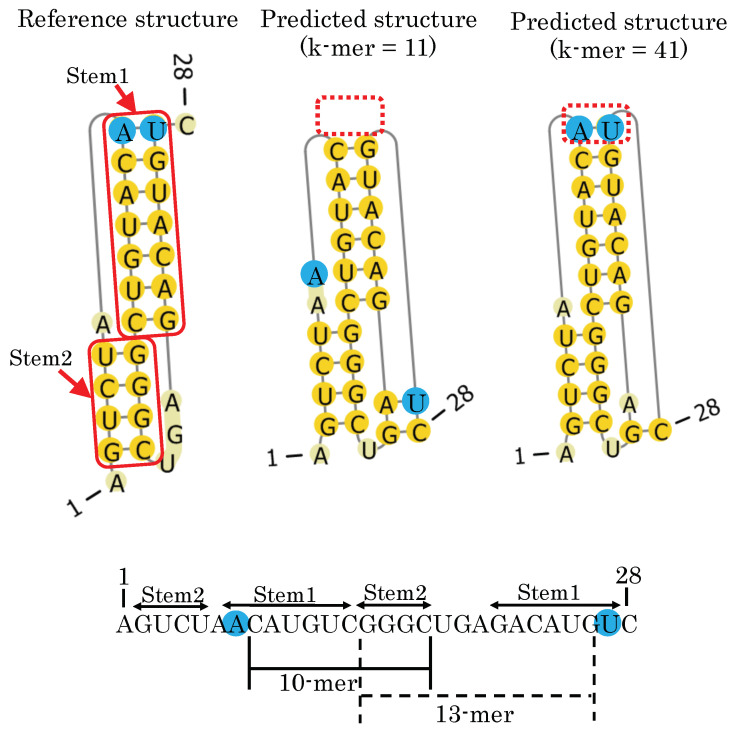
(**Top**) Comparison between the reference structure of ID PKB189 (**top-left**) and the predicted structures with context lengths *k* = 11 (**top-middle**) and *k* = 41 (**top-right**). (**Bottom**) Distance between two stems (Stem 1 and Stem 2) in the pseudoknotted structure.

The FNN model takes two *k*-mers around each base pair as input to infer its base-pairing probability, where *k* is the context length to model the length of loops and the contexts around the openings and closings of helices. As can be seen in Figure 7, different *k*-mer context lengths affect the prediction of pseudoknotted secondary structures. For example, consider the input bases when calculating the base-pairing probability of the blue-highlighted base pair (AU) using the FNN model. The FNN model with the context length *k* = 11 takes as input five bases in both the upstream and downstream directions from bases *i* and *j*. As seen in Figure 7 (bottom), the distances from bases A and U are 10 and 13 to Stem 2, respectively. This means that all the bases comprising Stem 2 are *not* completely located within the context length *k* = 11 around the base pair AU. On the other hand, for the FNN model with context length *k* = 41, all the bases of Stem 2 are completely located within the context around the base pair AU. This leads the FNN model to correctly predict the base pair AU, suggesting that a longer context length enables consideration of the dependency between stems in pseudoknotted substructures.

## 5. Conclusions

We propose a novel algorithm for directly inferring base-pairing probabilities with neural networks that enables us to accurately predict RNA secondary structures with pseudoknots. By combining IPknot-style decoding with the max-margin framework, our algorithm trains the model in the end-to-end manner to compute base-pairing probabilities under the constraints that RNA secondary structures, including pseudoknots, must satisfy. HotKnots 2.0 [32], on the other hand, finds a pseudoknotted secondary structure by using an MFE-based heuristic decoding algorithm with energy parameters of the Dirks–Pierce model or the Cao–Chen model trained on pseudoknotted reference structures. One of the advantages of our algorithm over HotKnots 2.0 is that no assumption about the architecture of RNA secondary structures is required. In other words, our model can be trained on arbitrary classes of pseudoknots, while HotKnots cannot be trained on more complicated classes of pseudoknots than the one assumed by the model. Furthermore, our algorithm can compute base-pairing probabilities, which can be used in various applications of RNA informatics, such as family classification [51,52], RNA–RNA interaction prediction [53] and simultaneous aligning and folding [54]. Accurate base-pairing probabilities calculated by our algorithm can improve the quality of such applications.

## Figures and Tables

**Table 1:** Accuracy of inferred base-pairing probabilities for TestSetB.

Implementation	Model	SEN	PPV	F
Neuralfold	BiRNN	0.649	0.601	0.624
Neuralfold	FNN	0.600	0.700	0.646
CentroidFold	McCaskill	0.513	0.544	0.528

**Table 2:** Accuracy of inferred base-pairing probabilities for the pk168 dataset.

Implementation	Model	SEN	PPV	F
Neuralfold	FNN	0.782±0.040	0.820±0.054	0.799±0.036
IPknot	McCaskill w/o refine.	0.619	0.710	0.661
IPknot	McCaskill w/refine.	0.753	0.684	0.717
IPknot	Dirks–Pierce	0.809	0.749	0.778

**Table 3:** Computation time for calculating base-pairing probabilities of sequences of various lengths.

ID	PKB229	PKB134	ASE_00193	CRW_00614	CRW_00774
Length (nt)	67	137	301	494	989
Neuralfold (FNN) IPknot	3.30 s	27.78 s	44.73 s	60.22 s	3 m 4.2 s
(w/o refine.)	0.01 s	0.05 s	0.18 s	0.55 s	2.64 s
(w/refine.)	0.03 s	0.08 s	0.31 s	1.03 s	5.86 s
(D&P)	8.36 s	9 m 4.7 s	n/a	n/a	n/a

Computation time was measured on an Intel Xeon E5-2680 (2.80 GHz) computer with 64 GB of memory and running Linux OS v2.6.32. FNN, feedforward neural network; D&P, Dirks–Pierce. IPknot with D&P failed to compute due to lack of memory for sequence lengths greater than 300.

## Data Availability

Not applicable.

## Abbreviations

The following abbreviations are used in this manuscript:

BiRNN	bi-directional recurrent neural network
FNN	feedforward neural network
MEA	maximum expected accuracy
MFE	minimum free energy
ncRNA	non-coding RNA
SSVM	structured support vector machine
